# Longitudinal Joint Modelling of Ordinal and Overdispersed Count Outcomes: A Bridge Distribution for the Ordinal Random Intercept

**DOI:** 10.1155/2021/5521881

**Published:** 2021-03-03

**Authors:** Payam Amini, Abbas Moghimbeigi, Farid Zayeri, Leili Tapak, Saman Maroufizadeh, Geert Verbeke

**Affiliations:** ^1^Department of Biostatistics and Epidemiology, School of Public Health, Ahvaz Jundishapur University of Medical Sciences, Ahvaz, Iran; ^2^Department of Biostatistics and Epidemiology, School of Health, Research Center for Health, Safety and Environment, Alborz University of Medical Sciences, Karaj, Iran; ^3^Department of Biostatistics, Member of Proteomics Research Center, School of Paramedical Sciences, Shahid Beheshti University of Medical Sciences, Tehran, Iran; ^4^Department of Biostatistics, School of Public Health, Hamadan University of Medical Science, Hamadan, Iran; ^5^School of Nursing and Midwifery, Guilan University of Medical Sciences, Rasht, Iran; ^6^Interuniversity Institute for Biostatistics and Statistical Bioinformatics, Katholieke Universiteit Leuven, B-3000 Leuven, Belgium; ^7^Interuniversity Institute for Biostatistics and Statistical Bioinformatics, Universiteit Hasselt, B-3590 Diepenbeek, Belgium

## Abstract

Associated longitudinal response variables are faced with variations caused by repeated measurements over time along with the association between the responses. To model a longitudinal ordinal outcome using generalized linear mixed models, integrating over a normally distributed random intercept in the proportional odds ordinal logistic regression does not yield a closed form. In this paper, we combined a longitudinal count and an ordinal response variable with Bridge distribution for the random intercept in the ordinal logistic regression submodel. We compared the results to that of a normal distribution. The two associated response variables are combined using correlated random intercepts. The random intercept in the count outcome submodel follows a normal distribution. The random intercept in the ordinal outcome submodel follows Bridge distribution. The estimations were carried out using a likelihood-based approach in direct and conditional joint modelling approaches. To illustrate the performance of the model, a simulation study was conducted. Based on the simulation results, assuming a Bridge distribution for the random intercept of ordinal logistic regression results in accurate estimation even if the random intercept is normally distributed. Moreover, considering the association between longitudinal count and ordinal responses resulted in estimation with lower standard error in comparison to univariate analysis. In addition to the same interpretation for the parameter in marginal and conditional estimates thanks to the assumption of a Bridge distribution for the random intercept of ordinal logistic regression, more efficient estimates were found compared to that of normal distribution.

## 1. Introduction

Many longitudinal studies are designed so that more than one response variable is recorded for the same subject. Thanks to various statistical tools, different types of outcomes such as count, nominal, and continuous can be analyzed jointly. For instance, patients after coronary artery bypass graft surgery are monitored several times a day, and their vital signs and bleeding condition are checked. In the case of kidney transplant data which is well explained in the following sections, the frequency of acute kidney transplant rejection as the count variable and estimated glomerular filtration rate as an ordinal variable are followed up for one year after the transplantation. In addition, the impact of several predictive variables such as patients' diabetes condition, antithymocyte globulin, urinary tract infection, and hypercalcemia on the response variables were assessed.

Univariate analysis of longitudinal outcomes has been developed by many authors during the last decades. The within-subject effect can be taken into account using several approaches such as covariance pattern model in generalized estimating equations or a random effect in subject-specific models [1–3]. Copula-based approaches have been utilized to account for the serial dependence of repeated observations [4, 5]. Various methods have been developed for jointly modeling longitudinal and survival data [6]. Kassahun et al. used generalized linear mixed models to model two longitudinal outcomes simultaneously. They considered weight and days of illness as the continuous and overdispersed count responses, respectively, [7]. Seyoum et al. considered the determinants of CD4 cell count change and adherence to highly active antiretroviral therapy among HIV adult patients, and they utilized a generalized linear mixed model to determine joint predictors of two longitudinal response variables over time. The adherence and CD4 cell were considered as ordinal and count outcomes respectively [8]. Joint modeling of discrete outcomes has been developed and discussed by Molenberghs and Verbeke. In addition to several approaches for jointly modeling two longitudinal responses, the association among the repeated measurements and between the two longitudinal processes can be considered via correlated random effects [9].

Copulas are widely used to integrate separate univariate regression models into a joint regression analysis of response variables [10, 11]. Copulas are very useful to combine two or more response variables from different types such as continuous, ordinal, and count [11, 12].

Several modeling approaches have been introduced to model clustered and hierarchical settings of response variables such as ordinal and overdispersed and/or zero inflated longitudinal count outcomes [13–15]. For count data, a Poisson distribution with a log-linear link function is commonly assumed. In the case of inequality of mean and variance of count response due to the extra heterogeneity, overdispersion would be considered in the modeling process. Negative binomial is a common choice to address this issue [16]. One of the key points in negative binomial regression, compared to other methods for analyzing overdispersed data, is that the mean is a single parameter. The negative binomial regression can be an extension of the Poisson-gamma model in which the mean in the Poisson distribution follows a gamma distribution in order to take the overdispersion into account [17, 18]. In the case of inflated zeros in the data, zero-inflated models are used [1, 19]. Analyzing methods for binary data have been extended to nominal and ordinal categorical outcomes [20].

Most commonly, a normally distributed random effect is considered for the ordinal outcome. In the case of a binary response, Wang and Louis showed that assuming a Bridge distribution for the random intercept in a logistic mixed model forces the fixed effects to have a marginal similar to conditional interpretation (conditional on the random intercepts) [21]. This idea was then applied by Lin et al. for evaluating the association between binary and continuous clustered data [22]. Bridge distribution is a useful technique for conditional assessment while allowing for meaningful marginal regression effects, especially for logistic regression. The key advantage of using Bridge distribution is that it allows the marginal probability of the binary response, integrated over the random intercept, to have a logistic structure with an odds ratio interpretation of the marginal regression effect. The regression parameters in the marginal logistic regression model are proportional to the corresponding regression parameters in the subject-specific conditional logistic model.

In this study, we aim to combine an ordinal and a possibly overdispersed count response variables by combining their correlated random intercepts. We assume a Bridge distribution for the ordinal outcome random intercept which is an extension of a hierarchical binary outcome. A Gaussian copula is used to integrate the two random intercepts [12]. This model can be fitted using commercially available statistical software, including the NLMIXED procedure of the Statistical Analysis System (SAS).

## 2. Materials and Methods

### 2.1. Notations and Distributions

For subject *i* at occasion *j*, let *y*_1*ij*_ represent the count response and *y*_2*ij*_is the ordinal response variable which can take the values 1, ⋯, *c*. The count response variable follows a Poisson distribution. When overdispersion is present, a negative binomial distribution with overdispersion parameter (*r*) and success probability (*p*) is used instead. The dispersion statistic can be determined as the ratio of the Pearson *χ*^2^ to its degrees of freedom and is a common method used to estimate overdispersion [23]. It has been argued that overdispersion exists if the dispersion statistic is over 1.2 and negative binomial outperforms simple Poisson model [24]. A logarithmic function is used to link the expected count response (log(*E*(*Y*_1*ij*_)) = log(*μ*_*ij*_)) to the systematic component ($X_{\overset{´}{ij}}\beta +w_{i}$) where $X_{\overset{´}{ij}}$ is the matrix of independent variables and *β* is the vector of corresponding coefficients. The random intercept of count response submodel (*w*_*i*_) follows a normal distribution (mean zero and variance *σ*_*w*_^2^). The ordinal response variable is assumed to follow multinomial distribution and *p*(*Y*_2*ij*_ ≤ *c*) is linked to a linear function of covariates ($\theta_{c}-X_{\overset{´}{ij}}\alpha -b_{i}$) via a traditional logit function. The random intercept of the ordinal response submodel (*b*_*i*_) follows a Bridge or normal distribution (mean zero and variance *σ*_*b*_^2^). In the case of two associated response variables, a correlation parameter (*ρ*) takes the association between the random intercepts into account. Details about the notations have been prepared in the Appendix.

### 2.2. Model Specification and Estimation

The two associated submodels are shown in equation (1) in which the mean count and ordinal response variables are linked to their systematic components through logarithm and logit functions. Let *p*_*c*_ = *p*(*Y*_2*ij*_ ≤ *c*), then the model can be specified as follows:

(1) $$\begin{array}{c} \left(\begin{array}{c} \text{Log}\left(\mu_{ij}\right)\\ \text{Logit}\left(p_{c}\right) \end{array}\right)=\left(\begin{array}{c} X_{\overset{´}{ı}j}\beta +w_{i}\\ \theta_{c}-X_{\overset{´}{ı}j}\alpha -b_{i} \end{array}\right). \end{array}$$

Bridge and normal distributions are considered for the random intercept of the ordinal and count outcome submodels. A copula approach is used in the case of different distributions to combine the random intercepts. The Bridge distribution proposed by Wang and Louis [21] allows both the conditional and marginal probabilities of the binary response to follow a logistic structure. Here, we have extended this assumption to an ordinal response variable. The Bridge distribution is well illustrated by Wang and Louis [21].

### 2.3. Bridge Distribution

Let *G*_*τ*_(*b*) be the cumulative Bridge distribution for subject-specific random effect *b* with the attenuation parameter *τ* and let *H*(.) be an inverse link function with the characteristics of being monotone, increasing, and twice differentiable.

(2) $$\begin{array}{c} \int H\left(X_{ij}^{\prime }\alpha +b_{i}\right)dG_{\tau \left(b\right)}=H\left(\tau X_{ij}^{\prime }\alpha +k\right). \end{array}$$

In equation (2), *k* is an unknown constant parameter and is equal to zero when the link unction is symmetric. To find the Bridge function, we need to use Fourier transformation (*ℱ*) and convolution operation. After differentiating and applying the convolution operation and Fourier transformation (*ℱ*) to equation (2), one can determine the general density function of Bridge distribution for any link function as shown in [3] where *H*′ = *h*.

(3) $$\begin{array}{c} g_{\tau }\left(b\right)=\int \frac{1}{2\pi }\exp\left(\text{i}\left(\frac{\text{k}}{\tau }-b\right)\text{u}\right)\frac{F_{\text{h}}\left(\text{u}/\tau \right)}{F_{\text{h}}\left(\text{u}\right)}\text{du}. \end{array}$$

In the case of logit link function, the density function of Bridge distribution is as [4]:

(4) $$\begin{array}{c} g_{\tau }\left(b\right)=\frac{1}{2\pi }\times \frac{\sin\left(\tau \pi \right)}{\cos\left(\pi \tau \right)+\cos h\left(\tau b\right)},\;0<\tau <1,-\infty <b<\infty . \end{array}$$

The mean and variance of the Bridge distribution are zero and (*π*^2^((1/*τ*^2^) − 1))/3, respectively. The intraclass correlation (ICC) can be determined by 1- *τ*.

The bridge density is symmetric with heavier tails and higher kurtosis than the normal density. The conditional $P\left(Y_{2ij}\leq c|X_{\overset{´}{ij}},b_{i}\right)$ and marginal $P\left(Y_{2ij}\leq c|X_{\overset{´}{ij}}\right)$ probabilities have the same logistic form. The parameter *τ* of the bridge distribution is the same for each subject which assures the exchangeability of subject random effects on the binary responses. More details about the Bridge distribution for binary response variables is well discussed in more details elsewhere [21, 22].

### 2.4. Gaussian Copula

Among many of copula families, Gaussian copula provides a convenient way to describe a complex relationship [25]. The Gaussian copula function is

(5) $$\begin{array}{c} C\left(X.Y;\rho \right)=\phi \left(\phi^{-1}\left(x\right).\phi^{-1}\left(y\right);\rho \right)=\int_{-\infty }^{\emptyset^{-1}\left(x\right)}\int_{-\infty }^{\emptyset^{-1}\left(y\right)}\frac{1}{2\pi \sqrt{1-\rho^{2}}}\left\{\frac{-\left(s^{2}-2\rho st+t^{2}\right)}{2\left(1-\rho^{2}\right)}\right\}dsdt, \end{array}$$

where *ϕ* standard normal cumulative distribution function. Let *z*_1_ and *z*_2_ follow a standard bivariate normal distribution so that *z*_2_ = *ϕ*_._^−1^(*G*(*b*)), and *z*_1_ = *ϕ*_._^−1^(*ϕ*(*w*)). Therefore, the bivariate distribution function of the random intercepts is

(6) $$\begin{array}{c} f\left(b_{i}.w_{i}\right)=\frac{1}{2\pi \sqrt{1-\rho^{2}}}e^{-\left(1/\left(2\left(1-\rho^{2}\right)\right)\right)\left({\left\{\phi_{\emptyset }^{-1}\left(1-\left(1/\pi \tau \right)\left[\left(\pi /2\right)-\arctan\left\{\left(e^{\tau b}+\cos\left(\pi \tau \right)\right)/\left(\sin\left(\pi \tau \right)\right)\right\}\right]\right)\right\}}^{2}+w^{2}-2\rho w\phi_{\emptyset }^{-1}\left(1-\left(1/\pi \tau \right)\left[\left(\pi /2\right)-\arctan\left\{\left(e^{\tau b}+\cos\left(\pi \tau \right)\right)/\left(\sin\left(\pi \tau \right)\right)\right\}\right]\right)\right)}. \end{array}$$

The variance matrix of two response variables can easily be seen to be given by

(7) $$\begin{array}{c} \text{Var}\left({\text{y}}_{\text{i}}\right)\sim \theta_{i}z_{i}Gz_{\overset{´}{ı}}\theta_{ı}+\gamma_{\text{i}}^{1/2}{\text{A}}_{\text{i}}^{1/2}{\text{R}}_{\text{i}}\left(\rho \right){\text{A}}_{\text{i}}^{1/2}\gamma_{\text{i}}^{1/2}=\left(\begin{array}{cc} \frac{r^{2}\left(1-p\right)}{p^{2}}\left[\frac{\left(1-p\right)}{p}\sigma_{w}^{2}+1\right] & \frac{r\left(1-p\right)}{p^{2}}\rho \sigma_{b}\sigma_{w}.p_{c}\left(1-p_{c}\right)\\ \frac{r\left(1-p\right)}{p^{2}}\rho \sigma_{b}\sigma_{w}.p_{c}\left(1-p_{c}\right) & p_{c}\left(1-p_{c}\right)\left[\sigma_{b}^{2}p_{c}\left(1-p_{c}\right)+1\right] \end{array}\right), \end{array}$$

where *γ*_*i*_is the diagonal overdispersion matrix, *A*_*i*_ is the diagonal variance matrix of response variables assuming zero random effects, and *R*_*i*_(*ρ*) (here an identity matrix) is the matrix denoting the correlation among residual errors.

(8) $$\begin{array}{c} {\left.\theta_{\text{i}}=\left(\frac{\delta \mu_{\text{i}}}{\delta \eta_{\text{i}}}\right)\right|}_{b=0}={\text{A}}_{i}=\left(\begin{array}{cc} \frac{r\left(1-p\right)}{p} & 0\\ 0 & p_{c}\left(1-p_{c}\right) \end{array}\right),\\ Z_{i}=\left[\begin{array}{cc} 1 & 0\\ 0 & 1 \end{array}\right],\\ G=\left(\begin{array}{cc} \sigma_{w}^{2} & \rho \sigma_{b}\sigma_{w}\\ \rho \sigma_{b}\sigma_{w} & \sigma_{b}^{2} \end{array}\right),\\ \gamma_{i}=\left(\begin{array}{cc} r & 0\\ 0 & 1 \end{array}\right),\\ R_{i}\left(\rho \right)=\left(\begin{array}{cc} 1 & 0\\ 0 & 1 \end{array}\right),\;\rho =1. \end{array}$$

### 2.5. Likelihood and Estimation

The likelihood function can be written as below where ∅ is the standard normal density function:

(9) $$\begin{array}{c} \overset{N}{\underset{i=1}{\prod }}\overset{n_{i}}{\underset{j=1}{\prod }}f\left(y_{1ij}\;|\;w_{i}.x_{ij}\right)f\left(y_{2ij}\;|\;b_{i}.x_{ij}\right)f\left(b_{i}.w_{i}\right)=\overset{N}{\underset{i=1}{\prod }}\overset{n_{i}}{\underset{j=1}{\prod }}\left\{\overset{c}{\underset{k=2}{\prod }}\left[\frac{e^{\theta_{k}-X_{\overset{´}{ı}j}\alpha -b_{i}}}{1+e^{\theta_{k}-X_{\overset{´}{ı}j}\alpha -b_{i}}}-\frac{e^{\theta_{k-1}-X_{\overset{´}{ı}j}\alpha -b_{i}}}{1+e^{\theta_{k-1}-X_{\overset{´}{ı}j}\alpha -b_{i}}}\right]\right\}\times \left\{{\left(\frac{\left(1/r\right)e^{X_{\overset{´}{ı}j}\beta +w_{i}}}{1+\left(1/r\right)e^{X_{\overset{´}{ı}j}\beta +w_{i}}}\right)}^{y_{1ij}}{\left(\frac{1}{1+\left(1/r\right)e^{X_{\overset{´}{ı}j}\beta +w_{i}}}\right)}^{r}\frac{\Gamma \left(y_{1ij}+r\right)}{\Gamma \left(y_{1ij}+1\right)\Gamma \left(r\right)}\right\}\times \frac{1}{2\pi \sqrt{1-\rho^{2}}}e^{\left(1/\left(2\left(1-\rho^{2}\right)\right)\right)\left({\left\{\emptyset_{\emptyset }^{-1}\left(1-\left(1/\pi \tau \right)\left[\left(\pi /2\right)-\arctan\left\{\left(e^{\tau b}+\cos\left(\pi \tau \right)\right)/\left(\sin\left(\pi \tau \right)\right)\right\}\right]\right)\right\}}^{2}+w^{2}-2\rho w\emptyset_{\emptyset }^{-1}\left(1-\left(1/\pi \tau \right)\left[\left(\pi /2\right)-\arctan\left\{\left(e^{\tau b}+\cos\left(\pi \tau \right)\right)/\left(\sin\left(\pi \tau \right)\right)\right\}\right]\right)\right)}. \end{array}$$

### 2.6. Real Data Application

We applied the proposed model to two read datasets in which associated count and ordinal response variables are assessed using several independent variables longitudinally.

### 2.7. Migraine Data

As a first example, we consider a prospective, two-arm, randomized, triple-blind, placebo-controlled trial in the neurology clinic of Shohadaye-Tajrish hospital, Tehran, Iran [26]. The patients were randomly divided into two equal groups, coriander fruit syrup and control (33 patients in each group). In addition to 500 mg of sodium valproate per day, the patients received either 15 mL of coriander fruit syrup or 15 mL of placebo syrup, three times a day, for a month, according to the code provided by the department of traditional pharmacy in Tehran University of Medical Sciences, Tehran, Iran. The subjects were measured at weeks 1, 2, 3, and 4. Each time, the mean severity of pain was evaluated, on a ten-point visual analog scale (VAS). Severity was categorized into three levels (0-0.30, 0.30-0.60, and 0.60-1) as the ordinal response. Moreover, the frequency of migraine attacks were recorded and used as the count response variable. The severity and frequency of migraine attacks were significantly associated at four time points, and hence, the joint modeling approach was utilized. At the end of each week, patients were referring to the neurology clinic to report the requested items. Time, intervention, and their interaction were considered as the predictive variables.

### 2.8. Kidney Transplant Data

In this historical cohort study, patients referred to the kidney transplant center of Urmia University of Medical Sciences from 2003 to 2014 were investigated [27]. Two main longitudinal response variables, the frequency of acute kidney transplant rejection as the count variable and estimated glomerular filtration rate as an ordinal variable in 5 levels, were assessed. These variables were recorded every 4 months after the transplantation, during one year. Glomerular filtration rate was estimated from abbreviated prediction equation provided by the Modification of Diet in Renal Disease study (MDRD). The stages were determined using the National Kidney Foundation (NKF) criteria as stage 1 with normal or high eGFR (eGFR > 90 mL/min), stage 2 mild chronic kidney disease (eGFR = 60 − 89 mL/min), stage 3 moderate chronic kidney disease (eGFR = 30 − 59 mL/min), stage 4 Severe chronic kidney disease (eGFR = 15 − 29 mL/min), and stage 5 end stage chronic kidney disease (eGFR < 15 mL/min) [28]. The independent and predictor variables were the type of kidney donor (relative/nonrelative), recipient's age and sex, anemia (yes/no), type of medication (Azathioprine/Cellcept/both/none), diabetes (yes/no), and antithymocyte globulin (yes/no), as well as complications after transplantation, such as proteinuria (yes/no), hyperkalemia (yes/no), hyperuricemia (yes/no), leukopenia (yes/no), myocardial infarction(yes/no), delayed graft function (yes/no), acute tubular necrosis (yes/no), urinary tract infection (yes/no), chronic allograft necrosis (yes/no), dyslipidemia (TG/CHOL), liver dysfunction (BIL-T/BIL-D), and hypercalcemia (yes/no).

### 2.9. Simulation Settings

A simulation study is conducted to assess the impact of different magnitudes of overdispersion, sample size, and the correlation between the random intercepts, in joint modelling of hierarchical ordinal and count outcome. Moreover, we assessed the difference in performance of the joint model regarding the distribution of (normal vs. Bridge) random intercept in the ordinal logistic regression submodel.

Bivariate normal distribution was used to generate associated normally distributed random intercept with (*ρ*) as the correlation coefficient. Gaussian copula was utilized to generate associated random intercepts where the associated random intercepts in the count and ordinal submodels follow normal and Bridge distributions, respectively. The correlations between the random intercepts (*ρ*) were chosen in three levels as zero (no correlation between the random intercepts), 0.4 (medium correlation between the random intercepts), and 0.8 (high correlation between the random intercepts). A continuous independent variable (time) was generated from uniform distribution (between 1 and 5) in 5 different points (5 occasions) so that the occasions differ for each subject, and time intervals are not equal. A binary independent variable (group) was generated from binomial distribution with 0.5 as the probability. The count response variable was generated from a negative binomial distribution with the probability equal to the exponential of its systematic component and three different negative binomial parameter values (1 as high, 5 as medium, and 150 as low overdispersion). The ordinal response variable with three levels (*θ*_1_: threshold 1, *θ*_2_: threshold 2) was generated from a multinomial distribution using the probabilities associated with logit link function. The model was fitted on three different sample sizes 50, 200, and 500. The models were specified as

(10) $$\begin{array}{c} \left(\begin{array}{c} \text{Log}\left(\mu_{ij}\right)\\ \text{Logit}\left(p_{c}\right) \end{array}\right)=\left(\begin{array}{c} \beta_{0}+\beta_{1}\text{grou}{\text{p}}_{\overset{´}{ı}}+\beta_{2}\text{tim}{\text{e}}_{\overset{´}{ı}j+}w_{i}\\ \theta_{c}-\alpha_{1}\text{grou}{\text{p}}_{\overset{´}{ı}}-\alpha_{2}\text{tim}{\text{e}}_{\overset{´}{ı}j}-b_{i} \end{array}\right),\\ \beta =\left(\beta_{0}.\beta_{1}.\beta_{2}\right)=\left(2.6.-0.6.-0.4\right),\\ \alpha =\left(\alpha_{1}.\alpha_{2}.\theta_{1}.\theta_{2}\right)=\left(0.6.0.1.-1.1\right),\\ \left(w_{i}.b_{i}\right)\sim {\text{Normal}}_{2}\text{ or Gaussian Copula}\left(\left(0.0\right).\left(\begin{array}{cc} 0.01 & \rho 0.13\\ \rho 0.13 & 1.7 \end{array}\right)\right). \end{array}$$

Moreover, and using the same generated datasets as explained above, the simulation results from the joint models were compared with separate univariate models to demonstrate the outperformance of joint modeling approach. The comparisons were made using the sets of data in which the sample sizes were 50 and 200 and the correlations between the random intercepts were chosen as zero, 0.4, and 0.8. Absolute value of bias ($\text{AVB}=\left|\bar{\theta }-\theta \right|$) and mean square error (MSE) were used to compare the estimated coefficients with their true values.

### 2.10. Computational Support

The R software version 3.3.1 was used to generate the datasets using several packages including “copula,” “bridgedist,” “MASS,” and “mnormt.” In addition, the NLMIXED procedure in SAS program version 9.2 was utilized to fit the proposed joint models.

## 3. Results

### 3.1. Migraine Data

Descriptive statistics of continuous and categorical characteristics of the two groups are described in details elsewhere [26]. The frequency of migraine attacks followed a Poisson distribution without any evidence of overdispersion and zero inflation. The longitudinal joint model was used to combine the frequency and severity of migraine attacks, and the results are shown in Table 1. However, the intensity of migraine attacks has reduced significantly over time; the coriander fruit syrup reduced the odds of higher stages of severity compared to the control group over time. Likelihood ratio test confirmed the use of random intercepts in the model. A significant association was found between the random effects (correlation = 0.392, *p* = 0.036) which confirms the use of a joint modeling approach. Based on the results, the odds of higher categories of migraine attack severity in the intervention group was 0.012 times than that of control group after each week. Moreover, the mean number of migraine attacks after each week in the intervention group was 0.28 less than that of control.

### 3.2. Kidney Transplant Data

The longitudinal joint model was used to combine the eGFR and frequency of rejections and the results are shown in Table 2. The frequency of rejections was overdispersed. The odds ratio of being in a higher stage of eGFR for one-year-older patient is 1.05 (*p* < 0.001). Females were less prone to experience a lower stage of eGFR compared to males. Being in a higher stage of eGFR for females was 2.42 times more than men. Patients without chronic allograft necrosis experienced higher stages of eGFR with the odds ratio of 0.44. The results exposes that time, DGF, ATN, and being diabetic did not affect eGFR. The acute rejection of kidney transplantation was assessed by ATG, DGF, UTI, CAN, liver dysfunction, and time. The expected number of rejections for a patient without ATG is 2.63 times the expected number of rejections for a patient with ATG (*p* = 0.048). In patients without hypercalcemia, the expected number of rejections would increase by a factor of 2.622. A positive correlation between eGFR stages and the frequency of acute kidney transplant rejection was observed. Moreover, the negative binomial parameter was significant. The results are shown in Table 2.

### 3.3. Simulation Results

Tables 3–8 show the simulation results. Regarding different sample sizes, different magnitude of overdispersion, and different amount of correlation between the random intercepts, the estimated values were almost close to the true values in various settings. Regarding AVB and MSE, the performance of the proposed joint model with the assumption of Bridge distribution for the random intercept of the ordinal outcome did not differ whether the random effect follows normal or Bridge distributions. The simulation results can be discussed in different aspects. We assumed three different scenarios for sample size, and the simulation results exposed that a sample size of 50 yields to considerable less accurate estimations than those of 200 and 500 subjects. In other words, considering 5 occasions for each subject, a total number of 250 observations was less efficient than that of 1000 and 2500. We considered three levels of dispersion as high, low, and medium. The higher the amount of negative binomial parameter, the bigger the estimated MSE and AVB. However, the large sample size decreases the amount of MSE and AVB in high value (low overdispersion). Generally and considering different scenarios, the magnitude of overdispersion did not affect the accuracy of the estimations after our proposed joint model. The standard deviation of the random intercepts and the estimated correlation coefficients between the random intercepts are very close to their true values with a relatively low MSE and AVB whether the random intercepts followed bivariate normal or Gaussian copula distributions. Moreover, the results were robust in different amount of correlation scenarios.

The results of comparing the joint and univariate models are presented in the Appendix. When the association between the random intercepts is high, the estimated coefficients in the joint modeling approach is significantly more robust than that of univariate approaches. The results when the sample is 50 is less accurate than that of 200. In the absence of correlation between the random intercepts, the estimations are almost the same for joint and univariate methods. For both of the joint and univariate approaches, our proposed model outputs reliable and accurate estimations whether the random intercept of the ordinal outcome was generated from normal or Bridge distributions.

## 4. Discussion

Longitudinal approaches consider the variation caused by the repeated measurements over the time. Two or more longitudinal response variables are frequently recorded in medical and clinical area. To combine the associated response variables, several approaches have been introduced and applied such as correlated random effects in a generalized linear mixed-effect model [1, 29, 30]. Primary studies were performed by Tate, and consequent researches have well discussed the conditional models as a major approach toward joint methods [31, 32]. Other approaches combine the responses directly [33]. In addition to GLMMs, the extension of Placket-Dale model to other mixed responses is straightforward and is well described elsewhere [29]. Besides, the generalizability of GLMM makes the extensions possible to other settings of combined discrete and continuous outcomes [1]. This article combined the ordinal and count outcomes through a bivariate distribution of their random intercepts. We assumed that the random intercept of the ordinal logistic regression follows Bridge distribution. Later, the normally distributed random intercept of the Poisson (negative binomial in case of overdispersion) was combined with the random intercept from the ordinal logistic submodel to take the association between the outcomes into account. Moreover, most of the studies assume that the random effects in a mixed model follow normal distribution, and we let the random intercepts follow Bridge and normal distributions. We compared the results of Bridge distribution with the frequently used assumption of normal. However, many of other distributions can be assumed based on the aim of research. The idea of using Bridge distribution for the random intercept of a longitudinal ordinal outcome can be used for both univariate and multivariate purposes.

In the case of a binary response variable, it had been shown that integrating over a normally distributed random intercept in a logistic model does not result in a closed form [22]. To make similar subject-specific and population average interpretations in terms of odds ratio, Bridge distribution was introduced [21]. In this paper, we have extended the use of Bridge distribution for the random intercept of binary logistic model to an ordinal proportional-odds cumulative logit model. Extra thresholds are estimated in the ordinal logistic model compared to the case of binary. The ordinal logistic regression requires some attentions due to its multiple categories in comparison to the case of binary. In the current study, the coefficients are interpreted in terms of proportional-odds cumulative logit model which is popular due to its relation to the concept of a continuous latent response variable [34].

The modeling of overdispersed count response was built upon several previous studies such as those of Molenberghs et al. in which normal and gamma random effects were used to take the subject-specific effect and overdispersion into account [35, 36]. Regarding the overdispersion, we assumed the count response to follow a negative binomial distribution which can be defined as a Poisson distribution with a gamma-distributed rate [17].

The simulation results showed that even if the random intercepts follow a bivariate normal distribution, assuming a Bridge distribution for the random intercept of the ordinal logistic submodel leads to estimations with lower standard error. Moreover, ignoring the association between the response variables resulted in estimations with higher MSE. It has been shown a rational association between the response variables.

In this study, we longitudinally combined ordinal and overdispersed count response variables in which the random intercepts follow Bridge and normal distributions, respectively. The random intercepts followed a bivariate normal copula. A maximum likelihood estimation approach was used to estimate the coefficients. The integration over the two random intercepts can be carried out via combination of analytical and numerical techniques. The SAS procedure NLMIXED is available for the estimation and integration processes.

## 5. Conclusion

Considering a Bridge distribution for the random intercept of ordinal logistic regression yields to accurate estimation even if the random intercept follows normal distribution. In the presence of any association between longitudinal count and ordinal responses, the estimations have lower standard error in comparison to univariate analysis.

## Figures and Tables

**Table 1:** The results of longitudinal joint modeling of severity and frequency of migraine attacks.

Variable	Estimate	Standard error	*p* value	95% CI		OR
*Ordinal process (severity)*						
Threshold 1	-6.293	0.957	<.0001	-8.169	-4.417	0.002
Threshold 2	-0.611	0.631	0.337	-1.848	0.626	0.543
Intervention	3.744	1.106	0.001	1.576	5.912	42.267
Week	-1.459	0.226	<0.001	-1.902	-1.016	0.232
Week^∗^intervention	-4.449	0.658	<0.001	-5.739	-3.159	0.012
Random effect standard deviation	0.967	0.170				
*Count process (frequency)*						
Intercept	0.854	0.106	<.001	0.646	1.062	2.349
Intervention	0.166	0.148	0.266	-0.124	0.456	1.181
Week	-0.196	0.043	<.001	-0.280	-0.112	0.822
Week^∗^intervention	-0.331	0.071	<.001	-0.470	-0.192	0.718
Random effect standard deviation	0.289	0.066				
Correlation between the random effects	0.392	0.203	0.058	-0.006	0.790	1.480

OR: odds ratio; CI: confidence interval.

**Table 2:** The results of longitudinal joint modeling of eGFR and the frequency of acute kidney transplant rejection.

Variable	Estimate	Standard error	*p* value	95% CI		OR
*Ordinal process (estimated glomerular filtration rate)*						
Age	0.047	0.010	<0.001	0.028	0.066	1.05
Sex (female)	0.884	0.278	0.002	0.335	1.434	2.42
Diabetes (no)	-0.195	0.288	0.499	-0.765	0.375	0.82
ATN (no)	0.346	0.696	0.620	-1.031	1.723	1.41
DGF (no)	2.140	1.231	0.084	-0.295	4.575	8.50
CAN (no)	-0.814	0.248	0.001	-1.305	-0.324	0.44
Time	-0.005	0.030	0.879	-0.064	0.055	0.99
Random effect standard deviation	0.322	0.041				
*Count process (acute kidney transplant rejection)*						
Sex (female)	-0.447	0.380	0.242	-1.200	0.306	0.640
ATG (no)	0.967	0.540	0.076	-0.102	2.035	2.630
Hypercalcemia (no)	0.964	0.536	0.075	-0.097	2.025	2.622
Proteinuria (no)	-0.641	0.393	0.105	-1.418	0.136	0.527
Hypertension (no)	-0.450	0.426	0.293	-1.294	0.393	0.638
Time	-0.046	0.054	0.389	-0.152	0.060	0.955
Random effect standard deviation	0.004	0.280				
Negative binomial parameter	0.172	0.045	<0.001	0.083	0.261	
Correlation between the random intercepts	0.765	0.101	<0.001	0.563	0.967	

ATG: antithymocyte globulin; DGF: delayed graft function; ATN: acute tubular necrosis; CAN: chronic allograft necrosis; OR: odds ratio; CI: confidence interval.

**Table 3:** Simulation study results with zero correlation between the random intercepts and normal distribution for the random intercept of the ordinal outcome.

		NBP = 1			NBP = 5			NBP = 150		
		Mean	AVB	MSE	Mean	AVB	MSE	Mean	AVB	MSE
*Count process*										
*β*_0_ = 2.6	50	2.6404	0.0404	0.0025	2.6398	0.0398	0.0025	2.5711	0.0289	0.0018
	200	2.6699	0.0699	0.0053	2.6120	0.0120	0.0006	2.5958	0.0042	0.0005
	500	2.6676	0.0676	0.0050	2.6105	0.0105	0.0005	2.6066	0.0066	0.0005
*β*_1_ = −0.6	50	-0.5734	0.0266	0.0007	-0.5750	0.0250	0.0006	-0.5936	0.0064	<0.0001
	200	-0.6200	0.0200	0.0004	-0.5838	0.0162	0.0003	-0.6057	0.0057	<0.0001
	500	-0.5948	0.0052	<0.0001	-0.5992	0.0008	<0.0001	-0.6060	0.0060	<0.0001
*β*_2_ = −0.4	50	-0.4382	0.0382	0.0016	-0.3689	0.0311	0.0010	-0.4213	0.0213	0.0005
	200	-0.4258	0.0258	0.0007	-0.4379	0.0379	0.0014	-0.3831	0.0169	0.0003
	500	-0.4234	0.0234	0.0006	-0.3847	0.0153	0.0002	-0.3908	0.0092	0.0001
*NBP*	50	1.0265	0.0265	0.0007	5.5933	0.5933	0.3520	150.7136	0.7136	0.5093
	200	1.0747	0.0747	0.0176	5.2590	0.2590	0.0672	150.7340	0.7340	0.5389
	500	1.0180	0.0180	0.0004	5.0060	0.0060	<0.0001	150.7735	0.7735	0.5983
*σ*_*w*_ = 0.1	50	0.0734	0.0266	0.0007	0.0780	0.0220	0.0005	0.0752	0.0248	0.0006
	200	0.0868	0.0132	0.0003	0.0786	0.0214	0.0005	0.0795	0.0205	0.0004
	500	0.0981	0.0019	0.0001	0.0912	0.0088	0.0001	0.0893	0.0107	0.0002
*Ordinal process*										
*α*_1_ = 0.6	50	0.6346	0.0346	0.0017	0.626	0.0260	0.0007	0.6378	0.0378	0.0014
	200	0.6318	0.0318	0.0012	0.6277	0.0277	0.0008	0.6216	0.0216	0.0005
	500	0.6214	0.0214	0.0005	0.6223	0.0223	0.0005	0.6195	0.0195	0.0004
*α*_2_ = 0.1	50	0.1532	0.0532	0.0028	0.1318	0.0318	0.0010	0.1214	0.0214	0.0005
	200	0.1376	0.0376	0.0014	0.1156	0.0156	0.0002	0.1135	0.0135	0.0002
	500	0.1222	0.0222	0.0008	0.1158	0.0158	0.0005	0.1102	0.0102	0.0003
*θ*_1_ = −1	50	-0.896	0.1040	0.0111	-0.8816	0.1184	0.0140	-0.8804	0.1196	0.0143
	200	-0.8047	0.1953	0.0384	-0.8862	0.1138	0.0130	-0.8973	0.1027	0.0106
	500	-1.0318	0.0318	0.0010	-0.9325	0.0675	0.0046	-0.9311	0.0689	0.0047
*θ*_2_ = 1	50	1.0616	0.0616	0.0038	1.2476	0.2476	0.0613	1.1486	0.1486	0.0221
	200	1.1979	0.1979	0.0392	1.1629	0.1629	0.0265	1.1186	0.1186	0.0141
	500	0.9176	0.0824	0.0068	1.0201	0.0201	0.0004	1.0106	0.0106	0.0001
*σ*_*b*_ = 1.3	50	1.1629	0.1371	0.0188	1.1645	0.1355	0.0184	1.1639	0.1361	0.0185
	200	1.1924	0.1076	0.0116	1.1945	0.1055	0.0112	1.1915	0.1085	0.0118
	500	1.2423	0.0577	0.0033	1.2745	0.0255	0.0007	1.2525	0.0475	0.0023
*ρ* = 0	50	0.0542	0.0542	0.0029	0.0666	0.0666	0.0045	0.0346	0.0346	0.0012
	200	0.0028	0.0028	<0.0001	0.0257	0.0257	0.0007	0.0169	0.0169	0.0003
	500	0.0017	0.0017	<0.0001	0.0023	0.0023	<0.0001	0.0009	0.0009	<0.0001

NBP: negative binomial parameter; AVB: the absolute value of biases; MSE: mean square error.

**Table 4:** Simulation study results with zero correlation between the random intercepts and Bridge distribution for the random intercept of the ordinal outcome.

		NBP = 1			NBP = 5			NBP = 150		
		Mean	AVB	MSE	Mean	AVB	MSE	Mean	AVB	MSE
*Count process*										
*β*_0_ = 2.6	50	2.7689	0.1689	0.0295	2.6292	0.0292	0.0018	2.5784	0.0216	0.0014
	200	2.6464	0.0464	0.0026	2.6114	0.0114	0.0006	2.6120	0.0120	0.0006
	500	2.6242	0.0242	0.0010	2.6107	0.0107	0.0005	2.6006	0.0006	0.0004
*β*_1_ = −0.6	50	-0.5870	0.0130	0.0002	-0.6024	0.0024	<0.0001	-0.6015	0.0015	<0.0001
	200	-0.6054	0.0054	<0.0001	-0.6023	0.0023	<0.0001	-0.6045	0.0045	<0.0001
	500	-0.5996	0.0004	<0.0001	-0.5986	0.0014	<0.0001	-0.5968	0.0032	<0.0001
*β*_2_ = −0.4	50	-0.3789	0.0211	0.0005	-0.3757	0.0243	0.0006	-0.3804	0.0196	0.0004
	200	-0.4297	0.0297	0.0009	-0.4285	0.0285	0.0008	-0.4055	0.0055	<0.0001
	500	-0.4231	0.0231	0.0005	-0.4116	0.0116	0.0001	-0.4034	0.0034	<0.0001
*NBP*	50	1.0230	0.0230	0.0006	5.3854	0.3854	0.1486	150.6349	0.6349	0.4031
	200	1.0060	0.0060	0.0002	5.0512	0.0512	0.0027	150.7227	0.7227	0.5224
	500	1.0010	0.0010	<0.0001	5.0036	0.0036	<0.0001	150.5830	0.5830	0.3399
*σ*_*w*_ = 0.1	50	0.1318	0.0318	0.0010	0.1304	0.0304	0.0009	0.0754	0.0246	0.0006
	200	0.1188	0.0188	0.0004	0.1255	0.0255	0.0007	0.0859	0.0141	0.0002
	500	0.1088	0.0088	0.0001	0.1106	0.0106	0.0002	0.0971	0.0029	<0.0001
*Ordinal process*										
*α*_1_ = 0.6	50	0.582	0.0180	0.0003	0.6082	0.0082	0.0001	0.6254	0.0254	0.0007
	200	0.5911	0.0089	0.0001	0.5823	0.0177	0.0003	0.6116	0.0116	0.0001
	500	0.5942	0.0058	<0.0001	0.6134	0.0134	0.0002	0.5925	0.0075	0.0001
*α*_2_ = 0.1	50	0.1428	0.0428	0.0018	0.0885	0.0115	0.0001	0.1243	0.0243	0.0006
	200	0.1466	0.0466	0.0022	0.0906	0.0094	0.0001	0.1181	0.0181	0.0003
	500	0.1158	0.0158	0.0005	0.0967	0.0033	0.0002	0.1088	0.0088	0.0003
*θ*_1_ = −1	50	-0.8847	0.1153	0.0133	-0.8949	0.1051	0.0111	-0.8568	0.1432	0.0205
	200	-0.8906	0.1094	0.0120	-0.8994	0.1006	0.0101	-0.9176	0.0824	0.0068
	500	-0.9401	0.0599	0.0036	-0.9301	0.0699	0.0049	-0.9395	0.0605	0.0037
*θ*_2_ = 1	50	1.1429	0.1429	0.0204	0.7435	0.2565	0.0658	0.8873	0.1127	0.0127
	200	1.0309	0.0309	0.0010	1.1412	0.1412	0.0199	1.1031	0.1031	0.0106
	500	1.0697	0.0697	0.0049	0.9123	0.0877	0.0077	0.9001	0.0999	0.0100
*σ*_*b*_ = 1.3	50	1.4381	0.1381	0.0191	1.4146	0.1146	0.0132	1.4271	0.1271	0.0162
	200	1.3915	0.0915	0.0084	1.3219	0.0219	0.0005	1.3616	0.0616	0.0038
	500	1.3734	0.0734	0.0054	1.3127	0.0127	0.0002	1.3212	0.0212	0.0005
*ρ* = 0	50	0.0476	0.0476	0.0023	0.0716	0.0716	0.0052	0.0419	0.0419	0.0018
	200	0.0134	0.0134	0.0002	0.0407	0.0407	0.0017	0.0181	0.0181	0.0003
	500	0.0059	0.0059	<0.0001	0.0007	0.0007	<0.0001	0.0009	0.0009	<0.0001

NBP: negative binomial parameter; AVB: the absolute value of biases; MSE: mean square error.

**Table 5:** Simulation study results with 0.4 correlation between the random intercepts and normal distribution for the random intercept of the ordinal outcome.

		NBP = 1			NBP = 5			NBP = 150		
		Mean	AVB	MSE	Mean	AVB	MSE	Mean	AVB	MSE
*Count process*										
*β*_0_ = 2.6	50	2.5794	0.0206	0.0004	2.5807	0.0193	0.0013	2.5703	0.0297	0.0018
	200	2.6111	0.0111	0.0001	2.5926	0.0074	0.0005	2.5811	0.0189	0.0008
	500	2.6012	0.0012	<0.0001	2.6095	0.0095	0.0005	2.5976	0.0024	0.0004
*β*_1_ = −0.6	50	-0.5750	0.0250	0.0006	-0.5942	0.0058	<0.0001	-0.5816	0.0184	0.0003
	200	-0.5865	0.0135	0.0002	-0.5935	0.0065	<0.0001	-0.5894	0.0106	0.0001
	500	-0.6001	0.0001	<0.0001	-0.6007	0.0007	<0.0001	-0.6105	0.0105	0.0001
*β*_2_ = −0.4	50	-0.3998	0.0002	<0.0001	-0.3949	0.0051	0.0001	-0.3908	0.0092	0.0001
	200	-0.4098	0.0098	0.0001	-0.4010	0.0010	<0.0001	-0.3918	0.0082	0.0001
	500	-0.4058	0.0058	<0.0001	-0.3992	0.0008	<0.0001	-0.3927	0.0073	0.0001
*NBP*	50	1.1084	0.1084	0.0118	5.6202	0.6202	0.3847	151.6914	1.6914	2.8609
	200	1.0643	0.0643	0.0041	5.2325	0.2325	0.0542	151.6560	1.6560	2.7425
	500	1.0030	0.0030	<0.0001	5.1730	0.1730	0.0299	150.2080	0.2080	0.0433
*σ*_*w*_ = 0.1	50	0.0857	0.0143	0.0002	0.0877	0.0123	0.0002	0.0843	0.0157	0.0003
	200	0.0864	0.0136	0.0002	0.0820	0.0180	0.0003	0.0813	0.0187	0.0004
	500	0.0935	0.0065	<0.0001	0.0973	0.0027	<0.0001	0.0915	0.0085	0.0001
*Ordinal process*										
*α*_1_ = 0.6	50	0.6383	0.0383	0.0015	0.6499	0.0499	0.0025	0.6227	0.0227	0.0005
	200	0.6136	0.0136	0.0002	0.6351	0.0351	0.0012	0.6148	0.0148	0.0002
	500	0.6011	0.0011	<0.0001	0.6123	0.0123	0.0002	0.6003	0.0003	<0.0001
*α*_2_ = 0.1	50	0.1731	0.0731	0.0053	0.1318	0.0318	0.0010	0.1371	0.0371	0.0014
	200	0.1318	0.0318	0.0010	0.1573	0.0573	0.0033	0.1212	0.0212	0.0005
	500	0.1204	0.0204	0.0004	0.1397	0.0397	0.0018	0.1173	0.0173	0.0005
*θ*_1_ = −1	50	-0.8459	0.1541	0.0237	-0.8126	0.1874	0.0351	-0.8878	0.1122	0.0126
	200	-0.9202	0.0798	0.0064	-0.8358	0.1642	0.0270	-0.8997	0.1003	0.0101
	500	-0.9638	0.0362	0.0013	-0.9253	0.0747	0.0056	-0.9427	0.0573	0.0033
*θ*_2_ = 1	50	1.3781	0.3781	0.1430	1.2285	0.2285	0.0522	1.2613	0.2613	0.0683
	200	1.1699	0.1699	0.0289	1.0716	0.0716	0.0051	1.0512	0.0512	0.0026
	500	1.0852	0.0852	0.0073	1.0378	0.0378	0.0015	1.0626	0.0626	0.0039
*σ*_*b*_ = 1.3	50	1.1989	0.1011	0.0102	1.3293	0.0293	0.0009	1.2741	0.0259	0.0007
	200	1.2128	0.0872	0.0076	1.3199	0.0199	0.0004	1.2883	0.0117	0.0002
	500	1.2315	0.0685	0.0047	1.3114	0.0114	0.0001	1.2914	0.0086	0.0001
*ρ* = 0.4	50	0.4337	0.0337	0.0011	0.4108	0.0108	0.0001	0.4112	0.0112	0.0001
	200	0.4061	0.0061	<0.0001	0.4400	0.0400	0.0016	0.4165	0.0165	0.0003
	500	0.4622	0.0622	0.0039	0.4621	0.0621	0.0039	0.4479	0.0479	0.0023

NBP: negative binomial parameter; AVB: the absolute value of biases; MSE: mean square error.

**Table 6:** Simulation study results with 0.4 correlation between the random intercepts and Bridge distribution for the random intercept of the ordinal outcome.

		NBP = 1		MSE	NBP = 5		MSE	NBP = 150		MSE
		Mean	AVB		Mean	AVB		Mean	AVB	
*Count process*										
*β*_0_ = 2.6	50	2.6665	0.0665	0.0054	2.5899	0.0101	0.0010	2.5623	0.0377	0.0024
	200	2.6104	0.0104	0.0006	2.5942	0.0058	0.0005	2.5819	0.0181	0.0008
	500	2.6010	0.0010	0.0004	2.6027	0.0027	0.0004	2.5968	0.0032	0.0004
*β*_1_ = −0.6	50	-0.5838	0.0162	0.0003	-0.6040	0.0040	<0.0001	-0.6140	0.0140	0.0002
	200	-0.5929	0.0071	0.0001	-0.5980	0.0020	<0.0001	-0.6082	0.0082	0.0001
	500	-0.6048	0.0048	<0.0001	-0.6012	0.0012	<0.0001	-0.6032	0.0032	<0.0001
*β*_2_ = −0.4	50	-0.4115	0.0115	0.0002	-0.4047	0.0047	0.0001	-0.4113	0.0113	0.0002
	200	-0.4014	0.0014	<0.0001	-0.4015	0.0015	<0.0001	-0.3911	0.0089	0.0001
	500	-0.3994	0.0006	<0.0001	-0.4051	0.0051	<0.0001	-0.4027	0.0027	<0.0001
*NBP*	50	1.0445	0.0445	0.0020	5.3994	0.3994	0.1596	151.3485	1.3485	1.8185
	200	1.0127	0.0127	0.0003	4.9996	0.0004	0.0001	149.2178	0.7822	0.6120
	500	0.9992	0.0008	<0.0001	5.0914	0.0914	0.0084	150.2360	0.2360	0.0557
*σ*_*w*_ = 0.1	50	0.1491	0.0491	0.0024	0.1234	0.0234	0.0006	0.0994	0.0006	<0.0001
	200	0.1429	0.0429	0.0018	0.1162	0.0162	0.0003	0.0942	0.0058	<0.0001
	500	0.1157	0.0157	0.0003	0.1066	0.0066	0.0001	0.0970	0.0030	<0.0001
*Ordinal process*										
*α*_1_ = 0.6	50	0.6287	0.0287	0.0008	0.6343	0.0343	0.0012	0.5890	0.0110	0.0001
	200	0.6183	0.0183	0.0003	0.6325	0.0325	0.0011	0.5883	0.0117	0.0001
	500	0.6004	0.0004	<0.0001	0.6128	0.0128	0.0002	0.5972	0.0028	<0.0001
*α*_2_ = 0.1	50	0.1291	0.0291	0.0008	0.0854	0.0146	0.0002	0.0887	0.0113	0.0001
	200	0.1393	0.0393	0.0015	0.1248	0.0248	0.0006	0.0987	0.0013	<0.0001
	500	0.1112	0.0112	0.0003	0.1001	0.0001	0.0002	0.0922	0.0078	0.0003
*θ*_1_ = −1	50	-0.8001	0.1999	0.0400	-0.9252	0.0748	0.0056	-0.9035	0.0965	0.0093
	200	-0.8649	0.1351	0.0183	-0.9148	0.0852	0.0073	-0.9072	0.0928	0.0086
	500	-0.9451	0.0549	0.0030	-0.9989	0.0011	<0.0001	-0.9704	0.0296	0.0009
*θ*_2_ = 1	50	0.8383	0.1617	0.0261	0.8427	0.1573	0.0247	0.8608	0.1392	0.0194
	200	1.1821	0.1821	0.0332	0.8955	0.1045	0.0109	0.8312	0.1688	0.0285
	500	0.9985	0.0015	<0.0001	0.9424	0.0576	0.0033	0.9822	0.0178	0.0003
*σ*_*b*_ = 1.3	50	1.3552	0.0552	0.0031	1.3816	0.0816	0.0067	1.3911	0.0911	0.0083
	200	1.3940	0.0940	0.0089	1.3935	0.0935	0.0088	1.3925	0.0925	0.0086
	500	1.2904	0.0096	0.0001	1.3595	0.0595	0.0035	1.3013	0.0013	<0.0001
*ρ* = 0.4	50	0.4383	0.0383	0.0015	0.4171	0.0171	0.0003	0.4276	0.0276	0.0008
	200	0.4135	0.0135	0.0002	0.4253	0.0253	0.0007	0.4327	0.0327	0.0011
	500	0.4283	0.0283	0.0008	0.4218	0.0218	0.0005	0.4165	0.0165	0.0003

NBP: negative binomial parameter; AVB: the absolute value of biases; MSE: mean square error.

**Table 7:** Simulation study results with 0.8 correlation between the random intercepts and normal distribution for the random intercept of the ordinal outcome.

		NBP = 1		MSE	NBP = 5		MSE	NBP = 150		MSE
		Mean	AVB		Mean	AVB		Mean	AVB	
*Count process*										
*β*_0_ = 2.6	50	2.6422	0.0422	0.0027	2.6220	0.0220	0.0014	2.6250	0.0250	0.0016
	200	2.5805	0.0195	0.0008	2.6140	0.0140	0.0006	2.6076	0.0076	0.0005
	500	2.6019	0.0019	0.0004	2.6024	0.0024	0.0004	2.6038	0.0038	0.0004
*β*_1_ = −0.6	50	-0.5816	0.0184	0.0003	-0.6113	0.0113	0.0001	-0.5882	0.0118	0.0001
	200	-0.5841	0.0159	0.0003	-0.6058	0.0058	<0.0001	-0.5930	0.0070	<0.0001
	500	-0.6006	0.0006	<0.0001	-0.6016	0.0016	<0.0001	-0.5998	0.0002	<0.0001
*β*_2_ = −0.4	50	-0.4181	0.0181	0.0004	-0.3991	0.0009	<0.0001	-0.4105	0.0105	0.0001
	200	-0.3950	0.0050	<0.0001	-0.4099	0.0099	0.0001	-0.4220	0.0220	0.0005
	500	-0.3840	0.0160	0.0003	-0.4070	0.0070	<0.0001	-0.4050	0.0050	<0.0001
*NBP*	50	1.0810	0.0810	0.0066	5.5362	0.5362	0.2875	148.4848	1.5152	2.2959
	200	1.0517	0.0517	0.0028	5.4830	0.4830	0.2334	151.0433	1.0433	1.0886
	500	1.0040	0.0040	<0.0001	5.5855	0.5855	0.3428	150.8672	0.8672	0.7520
*σ*_*w*_ = 0.1	50	0.0863	0.0137	0.0002	0.0871	0.0129	0.0002	0.0830	0.0170	0.0003
	200	0.0892	0.0108	0.0001	0.0831	0.0169	0.0003	0.0861	0.0139	0.0002
	500	0.0987	0.0013	<0.0001	0.0967	0.0033	0.0001	0.0918	0.0082	0.0001
*Ordinal process*										
*α*_1_ = 0.6	50	0.6606	0.0606	0.0037	0.6255	0.0255	0.0007	0.6603	0.0603	0.0036
	200	0.6290	0.0290	0.0008	0.6231	0.0231	0.0005	0.6624	0.0624	0.0039
	500	0.6055	0.0055	<0.0001	0.6020	0.0020	<0.0001	0.6294	0.0294	0.0009
*α*_2_ = 0.1	50	0.1803	0.0803	0.0064	0.1657	0.0657	0.0043	0.1645	0.0645	0.0042
	200	0.1720	0.0720	0.0052	0.1615	0.0615	0.0038	0.1682	0.0682	0.0047
	500	0.1693	0.0693	0.0050	0.1401	0.0401	0.0018	0.1512	0.0512	0.0028
*θ*_1_ = −1	50	-0.8495	0.1505	0.0227	-0.8917	0.1083	0.0117	-0.9167	0.0833	0.0070
	200	-0.9289	0.0711	0.0051	-0.8916	0.1084	0.0118	-0.9195	0.0805	0.0065
	500	-0.9529	0.0471	0.0022	-0.9676	0.0324	0.0011	-0.9785	0.0215	0.0005
*θ*_2_ = 1	50	1.3728	0.3728	0.1390	1.3594	0.3594	0.1292	1.2142	0.2142	0.0459
	200	1.1717	0.1717	0.0295	1.1962	0.1962	0.0385	1.0980	0.0980	0.0096
	500	1.1056	0.1056	0.0112	1.0246	0.0246	0.0006	1.0090	0.0090	0.0001
*σ*_*b*_ = 1.3	50	1.1966	0.1034	0.0107	1.2818	0.0182	0.0004	1.2796	0.0204	0.0004
	200	1.2019	0.0981	0.0097	1.2015	0.0985	0.0097	1.2908	0.0092	0.0001
	500	1.2260	0.0740	0.0055	1.2222	0.0778	0.0061	1.2923	0.0077	0.0001
*ρ* = 0.8	50	0.7068	0.0932	0.0087	0.7193	0.0807	0.0065	0.8492	0.0492	0.0024
	200	0.7167	0.0833	0.0070	0.8135	0.0135	0.0002	0.7834	0.0166	0.0003
	500	0.7297	0.0703	0.0049	0.7711	0.0289	0.0008	0.8293	0.0293	0.0009

NBP: negative binomial parameter; AVB: the absolute value of biases; MSE: mean square error.

**Table 8:** Simulation study results with 0.8 correlation between the random intercepts and Bridge distribution for the random intercept of the ordinal outcome.

		NBP = 1		MSE	NBP = 5		MSE	NBP = 150		MSE
		Mean	AVB		Mean	AVB		Mean	AVB	
*Count process*										
*β*_0_ = 2.6	50	2.5839	0.0161	0.0012	2.5893	0.0107	0.0011	2.5819	0.0181	0.0013
	200	2.6180	0.0180	0.0008	2.5957	0.0043	0.0005	2.6011	0.0011	0.0004
	500	2.6078	0.0078	0.0005	2.6011	0.0011	0.0004	2.6008	0.0008	0.0004
*β*_1_ = −0.6	50	-0.5976	0.0024	<0.0001	-0.5974	0.0026	<0.0001	-0.5949	0.0051	<0.0001
	200	-0.6018	0.0018	<0.0001	-0.5971	0.0029	<0.0001	-0.6031	0.0031	<0.0001
	500	-0.5979	0.0021	<0.0001	-0.5997	0.0003	<0.0001	-0.5991	0.0009	<0.0001
*β*_2_ = −0.4	50	-0.3636	0.0364	0.0014	-0.3810	0.0190	0.0004	-0.3952	0.0048	0.0001
	200	-0.4123	0.0123	0.0002	-0.3844	0.0156	0.0002	-0.4105	0.0105	0.0001
	500	-0.3466	0.0534	0.0029	-0.3863	0.0137	0.0002	-0.3981	0.0019	<0.0001
*NBP*	50	1.0100	0.0100	0.0001	5.5579	0.5579	0.3113	152.8764	2.8764	8.2737
	200	0.9912	0.0088	0.0002	5.0522	0.0522	0.0028	151.9130	1.9130	3.6597
	500	1.0010	0.0010	<0.0001	4.9405	0.0595	0.0035	150.9005	0.9005	0.8109
*σ*_*w*_ = 0.1	50	0.1291	0.0291	0.0009	0.1240	0.0240	0.0006	0.0852	0.0148	0.0002
	200	0.1210	0.0210	0.0004	0.1273	0.0273	0.0008	0.0892	0.0108	0.0001
	500	0.1033	0.0033	0.0001	0.1088	0.0088	0.0001	0.0945	0.0055	0.0001
*Ordinal process*										
*α*_1_ = 0.6	50	0.6450	0.0450	0.0020	0.6208	0.0208	0.0004	0.6439	0.0439	0.0019
	200	0.5931	0.0069	<0.0001	0.5853	0.0147	0.0002	0.5861	0.0139	0.0002
	500	0.5989	0.0011	<0.0001	0.6098	0.0098	0.0001	0.6001	0.0001	<0.0001
*α*_2_ = 0.1	50	0.0982	0.0018	<0.0001	0.1657	0.0657	0.0043	0.1239	0.0239	0.0006
	200	0.1429	0.0429	0.0018	0.1528	0.0528	0.0028	0.1117	0.0117	0.0001
	500	0.1207	0.0207	0.0006	0.1178	0.0178	0.0005	0.1036	0.0036	0.0002
*θ*_1_ = −1	50	-0.8440	0.1560	0.0243	-0.8705	0.1295	0.0168	-0.8993	0.1007	0.0102
	200	-0.9305	0.0695	0.0048	-0.9355	0.0645	0.0042	-0.9409	0.0591	0.0035
	500	-0.9266	0.0734	0.0054	-0.9940	0.0060	<0.0001	-0.9885	0.0115	0.0001
*θ*_2_ = 1	50	0.8827	0.1173	0.0138	0.8660	0.1340	0.0180	0.8386	0.1614	0.0261
	200	0.9797	0.0203	0.0004	0.8951	0.1049	0.0110	0.8671	0.1329	0.0177
	500	1.0117	0.0117	0.0002	0.9452	0.0548	0.0030	1.0256	0.0256	0.0007
*σ*_*b*_ = 1.3	50	1.3427	0.0427	0.0018	1.3512	0.0512	0.0026	1.2490	0.0510	0.0026
	200	1.3840	0.0840	0.0071	1.3740	0.0740	0.0055	1.2982	0.0018	<0.0001
	500	1.3820	0.0820	0.0067	1.3769	0.0769	0.0059	1.2994	0.0006	<0.0001
*ρ* = 0.8	50	0.7599	0.0401	0.0016	0.7888	0.0112	0.0001	0.8147	0.0147	0.0002
	200	0.8051	0.0051	<0.0001	0.8197	0.0197	0.0004	0.8189	0.0189	0.0004
	500	0.8195	0.0195	0.0004	0.7749	0.0251	0.0006	0.7997	0.0003	<0.0001

NBP: negative binomial parameter; AVB: the absolute value of biases; MSE: mean square error.

## Data Availability

The data is available on request contacting Dr. Payam Amini via email: payam.amini87@gmail.com.

## Supplementary Materials

Supplementary Materials The Appendix section includes three sections as A: SAS codes for the joint model; B: simulation results to compare the efficiency of estimation from the joint and univariate models; and C: notations in the text. Appendix A describes the NLMIXED procedure for our proposed model. Appendix B includes Tables 1 and 2 in which the results of comparing the joint and univariate models are presented for 50 and 200 sample sizes, respectively. Appendix C describes the notations used in this manuscript.
