# Supplementary material for: Longitudinal Joint Modelling of Ordinal and Overdispersed Count Outcomes: A Bridge Distribution for the Ordinal Random Intercept
Source: Comput Math Methods Med. 2021 Mar 3;2021:5521881. doi: 10.1155/2021/5521881 (PMC7946459; doi:10.1155/2021/5521881)
Supplement: Supplementary Materials — The Appendix section includes three sections as A: SAS codes for the joint model; B: simulation results to compare the efficiency of estimation from the joint and univariate models; and C: notations in the text. Appendix A describes the NLMIXED procedure for our proposed model. Appendix B includes Tables 1 and 2 in which the results of comparing the joint and univariate models are presented for 50 and 200 sample sizes, respectively. Appendix C describes the notations used in this manuscript. [file 5521881.f1.docx]

**Appendix (A). SAS codes for the joint model**

proc sort data=Data;

by pair;

run;

PROC NLMIXED gconv=0 data=Data qpoints=10 tech=NMSIMP;

PARMS C0=2.6 C1=-0.6 C2=-0.4 O1=0.5 O2=0.1 THRES1=-1 THRES2=1 tauo=1.3 tauc=0.1 rhobw=0.5 size=30;

if var="ordinal" then do;

pi=constant("pi");

uo=probnorm(b);

phi=1.0/sqrt(1+tauo*tauo*(3/pi*pi));

U=(1/phi)*log(sin(pi*uo*phi)/sin(phi*pi*(1-uo)));

Z = O1*group1+O2*time1 + U;

IF (res=0) THEN P = 1 / (1 + EXP(-(THRES1-Z)));

ELSE IF (res=1) THEN P = (1/(1 + EXP(-(THRES2-Z)))) - (1/(1 + EXP(-(THRES1-Z))));

ELSE IF (res=2) THEN P = 1 - (1 / (1 + EXP(-(THRES2-Z))));

LL= LOG(P);

END;

if var="count" then do;

m1=C0+C1*group1+C2*time1+z1*tauc;

mu=exp(m1);

LL=lgamma(res+size)-lgamma(res+1)-lgamma(size) + res*log((1/size)*mu)-(res+size)*log(1+(1/size)*mu);

END;

MODEL res ~ GENERAL(LL);

RANDOM b z1 ~ NORMAL([0,0],[1,rhobw,1]) SUBJECT=id1;

ods output ParameterEstimates=pars;

ods output FitStatistics=aic;

RUN;

**Appendix (B). Simulation results to compare the efficiency of estimation from the joint and univariate models**

**Table 1.** Simulation results to compare the efficiency of estimation from the joint and univariate models with 200 sample size

|  |  |  | | |  | **Association** | | |  |  | | |
| --- | --- | --- | --- | --- | --- | --- | --- | --- | --- | --- | --- | --- |
|  |  | **0** | | |  | **0.4** | | |  | **0.8** | | |
| **Count Process** | | **Mean** | **AVB** | **MSE** |  | **Mean** | **AVB** | **MSE** |  | **Mean** | **AVB** | **MSE** |
| $C_{0}$=2.6 | J-NN | 2.6699 | 0.0699 | 0.0053 |  | 2.6111 | 0.0111 | 0.0001 |  | 2.5805 | 0.0195 | 0.0008 |
|  | J-NB | 2.6464 | 0.0464 | 0.0026 |  | 2.6104 | 0.0104 | 0.0006 |  | 2.6180 | 0.0180 | 0.0008 |
|  | S-NN | 2.9242 | 0.3242 | 0.0087 |  | 2.5781 | 0.0219 | 0.0153 |  | 2.5227 | 0.0773 | 0.0200 |
|  | S-NB | 2.6044 | 0.0044 | 0.0008 |  | 2.5035 | 0.0965 | 0.0245 |  | 2.4589 | 0.1411 | 0.0314 |
| $C_{1}$=-0.6 | J-NN | -0.6200 | 0.0200 | 0.0004 |  | -0.5865 | 0.0135 | 0.0002 |  | -0.5841 | 0.0159 | 0.0003 |
|  | J-NB | -0.6054 | 0.0054 | <0.0001 |  | -0.5929 | 0.0071 | 0.0001 |  | -0.6018 | 0.0018 | <0.0001 |
|  | S-NN | -0.6104 | 0.0104 | 0.0019 |  | -0.6043 | 0.0043 | 0.0018 |  | -0.6042 | 0.0042 | 0.0009 |
|  | S-NB | -0.5968 | 0.0032 | 0.0013 |  | -0.5940 | 0.0060 | 0.0013 |  | -0.6059 | 0.0059 | 0.0023 |
| $C_{2}$=-0.4 | J-NN | -0.4258 | 0.0258 | 0.0007 |  | -0.4098 | 0.0098 | 0.0001 |  | -0.3950 | 0.0050 | <0.0001 |
|  | J-NB | -0.4297 | 0.0297 | 0.0009 |  | -0.4014 | 0.0014 | <0.0001 |  | -0.4123 | 0.0123 | 0.0002 |
|  | S-NN | -0.5055 | 0.1055 | 0.0045 |  | -0.3929 | 0.0071 | 0.0017 |  | 0.3076 | 0.2076 | 0.04314 |
|  | S-NB | -0.4023 | 0.0023 | 0.0023 |  | -0.3705 | 0.0295 | 0.0025 |  | -0.3540 | 0.0460 | 0.0033 |
| $\sigma_{c}$=0.1 | J-NN | 0.0868 | 0.0132 | 0.0003 |  | 0.0864 | 0.0136 | 0.0002 |  | 0.0892 | 0.0108 | 0.0001 |
|  | J-NB | 0.1188 | 0.0188 | 0.0004 |  | 0.1429 | 0.0429 | 0.0018 |  | 0.1210 | 0.0210 | 0.0004 |
|  | S-NN | 0.1146 | 0.0146 | 0.0019 |  | 0.3095 | 0.2029 | 0.0438 |  | 0.3076 | 0.2076 | 0.0431 |
|  | S-NB | 0.0857 | 0.0143 | 0.0083 |  | 0.0732 | 0.0268 | 0.0078 |  | 0.0812 | 0.0188 | 0.0064 |
| **Ordinal process** | |  |  |  |  |  |  |  |  |  |  |  |
| $O_{1}$=0.6 | J-NN | 0.6318 | 0.0318 | 0.0012 |  | 0.6136 | 0.0136 | 0.0002 |  | 0.6290 | 0.0290 | 0.0008 |
|  | J-NB | 0.5911 | 0.0089 | 0.0001 |  | 0.6183 | 0.0183 | 0.0003 |  | 0.5931 | 0.0069 | <0.0001 |
|  | S-NN | 0.5915 | 0.0085 | 0.0009 |  | 0.5979 | 0.0021 | 0.0017 |  | 0.5919 | 0.0081 | 0.0019 |
|  | S-NB | 0.6477 | 0.0477 | 0.0027 |  | 0.7672 | 0.1672 | 0.0286 |  | 0.6761 | 0.0761 | 0.0061 |
| $O_{2}$=0.1 | J-NN | 0.1376 | 0.0376 | 0.0014 |  | 0.1318 | 0.0318 | 0.0010 |  | 0.1720 | 0.0720 | 0.0032 |
|  | J-NB | 0.1466 | 0.0466 | 0.0022 |  | 0.1393 | 0.0393 | 0.0015 |  | 0.1429 | 0.0429 | 0.0018 |
|  | S-NN | 0.1519 | 0.0519 | 0.0083 |  | 0.2003 | 0.1003 | 0.0158 |  | 0.1207 | 0.0207 | 0.0062 |
|  | S-NB | 0.0538 | 0.0461 | 0.0027 |  | 0.2244 | 0.1244 | 0.0166 |  | 0.0636 | 0.0364 | 0.0059 |
| $\gamma_{1}$= -1 | J-NN | -0.8047 | 0.1953 | 0.0384 |  | -0.9202 | 0.0798 | 0.0064 |  | -0.9289 | 0.0711 | 0.0051 |
|  | J-NB | -0.8906 | 0.1094 | 0.0120 |  | -0.8649 | 0.1351 | 0.0183 |  | -0.9305 | 0.0695 | 0.0048 |
|  | S-NN | -0.7176 | 0.2824 | 0.1309 |  | -0.5753 | 0.4247 | 0.2328 |  | -0.8236 | 0.1764 | 0.0836 |
|  | S-NB | -1.0767 | 0.0767 | 0.0133 |  | -0.4914 | 0.5086 | 0.2684 |  | -1.0325 | 0.0325 | 0.0066 |
| $\gamma_{2}$=1 | J-NN | 1.1979 | 0.1979 | 0.0392 |  | 1.1699 | 0.1699 | 0.0289 |  | 1.1717 | 0.1717 | 0.0295 |
|  | J-NB | 1.0309 | 0.0309 | 0.0010 |  | 1.1821 | 0.1821 | 0.0332 |  | 0.9797 | 0.0203 | 0.0004 |
|  | S-NN | 0.9115 | 0.0885 | 0.0594 |  | 1.0625 | 0.0625 | 0.0560 |  | 0.8189 | 0.1811 | 0.0852 |
|  | S-NB | 0.6319 | 0.3681 | 0.1429 |  | 1.2248 | 0.2248 | 0.0603 |  | 0.6609 | 0.3391 | 0.1206 |
| $\sigma_{o}$=1.3 | J-NN | 1.1924 | 0.1076 | 0.0116 |  | 1.2128 | 0.0872 | 0.0076 |  | 1.2019 | 0.0981 | 0.0097 |
|  | J-NB | 1.3915 | 0.0915 | 0.0084 |  | 1.3940 | 0.0940 | 0.0089 |  | 1.3840 | 0.0840 | 0.0071 |
|  | S-NN | 1.3810 | 0.0810 | 0.0083 |  | 1.4210 | 0.1210 | 0.0163 |  | 0.4102 | 0.8898 | 0.7935 |
|  | S-NB | 1.3152 | 0.0152 | 0.0095 |  | 1.1959 | 0.1041 | 0.0109 |  | 1.6318 | 0.3318 | 0.1103 |

**J-NN**: Joint model with normal random intercept for the random intercept of the ordinal logistic submodel; **J-NB**: Joint model with Bridge random intercept for the random intercept of the ordinal logistic submodel; **S-NN**: Univariate model with normal random intercept for the random intercept of the ordinal logistic submodel; **S-NB**: Joint model with Bridge random intercept for the random intercept of the ordinal logistic submodel; **AVB**: Absolute value of bias; **MSE**: Mean Square Error

**Table 2.** Simulation results to compare the efficiency of estimation from the joint and univariate models with 200 sample size

|  |  |  | | |  | **Association** | | |  |  | | |
| --- | --- | --- | --- | --- | --- | --- | --- | --- | --- | --- | --- | --- |
|  |  | **0** | | |  | **0.4** | | |  | **0.8** | | |
|  |  | **میانگین** | **AVB** | **MSE** |  | **میانگین** | **AVB** | **MSE** |  | **میانگین** | **AVB** | **MSE** |
| **Count Process** | | |  |  |  |  |  |  |  |  |  |  |
| $C_{0}$=2.6 | J-NN | 2.6404 | 0.0404 | 0.0025 |  | 2.5794 | 0.0206 | 0.0004 |  | 2.6422 | 0.0422 | 0.0027 |
|  | J-NB | 2.7689 | 0.1689 | 0.0295 |  | 2.6665 | 0.0665 | 0.0054 |  | 2.5839 | 0.0161 | 0.0012 |
|  | S-NN | 2.6131 | 0.0131 | 0.0108 |  | 2.5372 | 0.0628 | 0.0157 |  | 2.5270 | 0.0730 | 0.0171 |
|  | S-NB | 2.6857 | 0.0857 | 0.0175 |  | 2.5652 | 0.0348 | 0.0126 |  | 2.4678 | 0.1322 | 0.0268 |
| $C_{1}$=-0.6 | J-NN | -0.5734 | 0.0266 | 0.0007 |  | -0.5750 | 0.0250 | 0.0006 |  | -0.5816 | 0.0184 | 0.0003 |
|  | J-NB | -0.5870 | 0.0130 | 0.0002 |  | -0.5838 | 0.0162 | 0.0003 |  | -0.5976 | 0.0024 | <0.0001 |
|  | S-NN | -0.5869 | 0.0131 | 0.0002 |  | -0.5980 | 0.0020 | 0.0011 |  | -0.6009 | 0.0009 | 0.0021 |
|  | S-NB | -0.6089 | 0.0089 | 0.0001 |  | -0.5955 | 0.0045 | 0.0023 |  | -0.6074 | 0.0074 | 0.0037 |
| $C_{2}$=-0.4 | J-NN | -0.4382 | 0.0382 | 0.0016 |  | -0.3998 | 0.0002 | <0.0001 |  | -0.4181 | 0.0181 | 0.0004 |
|  | J-NB | -0.3789 | 0.0211 | 0.0005 |  | -0.4115 | 0.0115 | 0.0002 |  | -0.3636 | 0.0364 | 0.0014 |
|  | S-NN | -0.3741 | 0.0259 | 0.0019 |  | -0.3783 | 0.0217 | 0.0018 |  | -0.3753 | 0.0247 | 0.0019 |
|  | S-NB | -0.4333 | 0.0333 | 0.0022 |  | -0.3931 | 0.0069 | 0.0013 |  | -0.3582 | 0.0417 | 0.0028 |
| $\sigma_{c}$=0.1 | J-NN | 0.0734 | 0.0266 | 0.0007 |  | 0.0857 | 0.0143 | 0.0002 |  | 0.0863 | 0.0137 | 0.0002 |
|  | J-NB | 0.1318 | 0.0318 | 0.0010 |  | 0.1491 | 0.0491 | 0.0024 |  | 0.1291 | 0.0291 | 0.0009 |
|  | S-NN | 0.2512 | 0.1512 | 0.0229 |  | 0.2431 | 0.1431 | 0.0205 |  | 0.2404 | 0.1404 | 0.0198 |
|  | S-NB | 0.0883 | 0.0117 | 0.0002 |  | 0.0834 | 0.0166 | 0.0183 |  | 0.0847 | 0.0153 | 0.0143 |
| **Ordinal Process** | |  |  |  |  |  |  |  |  |  |  |  |
| $O_{1}$=0.6 | J-NN | 0.6346 | 0.0346 | 0.0017 |  | 0.6383 | 0.0383 | 0.0015 |  | 0.6606 | 0.0606 | 0.0037 |
|  | J-NB | 0.582 | 0.0180 | 0.0003 |  | 0.6287 | 0.0287 | 0.0008 |  | 0.6450 | 0.0450 | 0.0020 |
|  | S-NN | 0.5827 | 0.0173 | 0.0010 |  | 0.6175 | 0.0175 | 0.0019 |  | 0.6433 | 0.0433 | 0.0039 |
|  | S-NB | 0.6752 | 0.0752 | 0.0059 |  | 0.6935 | 0.0935 | 0.0090 |  | 0.6640 | 0.0640 | 0.0044 |
| $O_{2}$=0.1 | J-NN | 0.1532 | 0.0532 | 0.0028 |  | 0.1731 | 0.0731 | 0.0053 |  | 0.1803 | 0.0803 | 0.0064 |
|  | J-NB | 0.1428 | 0.0428 | 0.0018 |  | 0.1291 | 0.0291 | 0.0008 |  | 0.0982 | 0.0018 | <0.0001 |
|  | S-NN | 0.2343 | 0.1343 | 0.0222 |  | 0.0821 | 0.0179 | 0.0143 |  | 0.0117 | 0.0883 | 0.0120 |
|  | S-NB | 0.2525 | 0.1525 | 0.0238 |  | 0.2776 | 0.1776 | 0.0323 |  | 0.2871 | 0.1871 | 0.0357 |
| $\gamma_{1}$= -1 | J-NN | -0.896 | 0.1040 | 0.0111 |  | -0.8459 | 0.1541 | 0.0237 |  | -0.8495 | 0.1505 | 0.0227 |
|  | J-NB | -0.8847 | 0.1153 | 0.0133 |  | -0.8001 | 0.1999 | 0.0400 |  | -0.8440 | 0.1560 | 0.0243 |
|  | S-NN | -0.4647 | 0.5353 | 0.3247 |  | -0.9366 | 0.0634 | 0.0895 |  | -1.1034 | 0.1034 | 0.0484 |
|  | S-NB | -0.4408 | 0.5592 | 0.3173 |  | -0.3543 | 0.6457 | 0.4238 |  | -0.3497 | 0.6503 | 0.4295 |
| $\gamma_{2}$=1 | J-NN | 1.0616 | 0.0616 | 0.0038 |  | 1.3781 | 0.3781 | 0.1430 |  | 1.3728 | 0.3728 | 0.1390 |
|  | J-NB | 1.1429 | 0.1429 | 0.0204 |  | 0.8383 | 0.1617 | 0.0261 |  | 0.8827 | 0.1173 | 0.0138 |
|  | S-NN | 1.1801 | 0.1801 | 0.0706 |  | 0.6889 | 0.3111 | 0.1522 |  | 0.5222 | 0.4778 | 0.2661 |
|  | S-NB | 1.3065 | 0.3065 | 0.0985 |  | 1.3916 | 0.3916 | 0.1602 |  | 1.3945 | 0.3945 | 0.1622 |
| $\sigma_{o}$=1.3 | J-NN | 1.1629 | 0.1371 | 0.0188 |  | 1.1989 | 0.1011 | 0.0102 |  | 1.1966 | 0.1034 | 0.0107 |
|  | J-NB | 1.4381 | 0.1381 | 0.0191 |  | 1.3552 | 0.0552 | 0.0031 |  | 1.3427 | 0.0427 | 0.0018 |
|  | S-NN | 1.5291 | 0.2291 | 0.0525 |  | 0.5298 | 0.7702 | 0.5933 |  | 1.3720 | 0.0720 | 0.0155 |
|  | S-NB | 1.1420 | 0.1580 | 0.0250 |  | 1.1472 | 0.1528 | 0.0234 |  | 1.1396 | 0.1604 | 0.0258 |

**J-NN**: Joint model with normal random intercept for the random intercept of the ordinal logistic submodel; **J-NB**: Joint model with Bridge random intercept for the random intercept of the ordinal logistic submodel; **S-NN**: Univariate model with normal random intercept for the random intercept of the ordinal logistic submodel; **S-NB**: Joint model with Bridge random intercept for the random intercept of the ordinal logistic submodel; **AVB**: Absolute value of bias; **MSE**: Mean Square Error

**Appendix C**: Notations in the text

| **Definition** | **Notation** |
| --- | --- |
| Indicates the subject ranges from 1 to N | i |
| Indicates the occasion in which the response variable is measured for subject i ranges from 1 to $n_{i}$ | j |
| Count response variable for subject i at occasion j | $y_{1ij}$ |
| Ordinal response variable for subject i at occasion j | $y_{2ij}$ |
| Overdispersion parameter in negative binomial distribution | r |
| success probability in negative binomial distribution | p |
| Expected count response for subject i at occasion j | $\mu_{ij}$ |
| Matrix of independent variables for subject i at occasion j | $X_{\acute{ij}}$ |
| Vector of corresponding coefficients in the count response submodel | $\beta$ |
| Vector of corresponding coefficients in the ordinal response submodel | $\alpha$ |
| Subject specific random intercept in the count response submodel | $w_{i}$ |
| Subject specific random intercept in the ordinal response submodel | $b_{i}$ |
| Variance of subject specific random intercept in the count response submodel | $\sigma_{w}^{2}$ |
| Variance of subject specific random intercept in the ordinal response submodel | $\sigma_{b}^{2}$ |
| The level of ordinal response variable | c |
| The c^th^ threshold in the ordinal logistic regression submodel | $\theta_{c}$ |
| The coefficient of correlation between the random intercepts $w_{i}$ and $b_{i}$ | ρ |
| $p(Y_{2ij}\leq c)$ | $p_{c}$ |
| Any cumulative inverse link function | $H$ |
| Any inverse link function | $h$=$H^{'}$ |
| Attenuation parameter in the Bridge distribution | $\tau$ |
| Bridge distribution for subject specific random effect $b_{i}$ with the parameter$\tau$ | $G_{\tau}\left( b \right)$ |
| unknown constant parameters in the Bridge distribution | k |
| The Fourier transformation | $\mathcal{F}$ |
| Gaussian cumulative distribution function | $\phi$ |
| An equation to find the combined the Gaussian copula of $w_{i}$ and $b_{i}$ | $z_{1}=\phi_{.}^{-1}\left( \phi\left( w \right) \right)$ |
| An equation to find the combined the Gaussian copula of $w_{i}$ and $b_{i}$ | $z_{2}=\phi_{.}^{-1}\left( G\left( b \right) \right)$ |
| the diagonal overdispersion matrix | $\gamma_{i}$ |
| the diagonal variance matrix of response variables assuming zero random effects | $A_{i}$ |
| the matrix denoting the correlation among residual errors | $R_{i}(\rho)$ |
